# Content validity and meaningful change for the FACIT-Fatigue scale in warm autoimmune hemolytic anemia: results from qualitative interview studies with patients

**DOI:** 10.1186/s41687-025-00930-0

**Published:** 2025-07-29

**Authors:** Sheryl Pease, Rikki Mangrum, Karolina Schantz, Christina Slota, Lindsey Rubin, Susan Martin, Cathye Shu, Kayla Scippa

**Affiliations:** 1 Johnson & Johnson, Patient Reported Outcomes, Raritan, NJ USA; 2 Vector Psychometric Group, LLC, Chapel Hill, NC USA; 3 RTI Health Solutions, Patient-Centered Outcomes Assessment, Research Triangle Park, NC USA; 4 Johnson & Johnson, Immunology, Spring House, PA USA

**Keywords:** Warm autoimmune hemolytic anemia, FACIT-Fatigue, Concept elicitation, Cognitive debriefing, Rare disease, Patient-reported outcomes

## Abstract

**Purpose:**

Warm autoimmune hemolytic anemia (wAIHA) is a rare disorder characterized by premature destruction of red blood cells (hemolysis) and fatigue that affects patients’ health-related quality of life. This study aimed to identify fatigue concepts important to patients and evaluate the content validity of the Functional Assessment of Chronic Illness Therapy-Fatigue Scale (FACIT-Fatigue) in the wAIHA patient population.

**Methodology:**

Two observational studies collected data via 60-minute, individual interviews. Eligible participants were English-speaking, US-resident adults diagnosed with wAIHA by a physician. Both studies gathered data regarding fatigue experiences that mattered to patients and comprehension and relevance of the FACIT-Fatigue; one study also gathered data about patient perspectives on meaningful change in item-level response selection.

**Results:**

Each study enrolled 10 individuals with wAIHA (*n* = 20). All participants described fatigue as the most prominent or most bothersome symptom, with substantial impacts on daily functioning, family and social life, and emotional well-being. The FACIT-Fatigue was well understood, comprehensive, and relevant to participants’ experiences with wAIHA. Data analysis indicated that at least a 3-point change in total score likely reflects a meaningful change in fatigue.

**Conclusions:**

Fatigue is the primary patient-reported wAIHA symptom and important to measure from the patient perspective. Findings demonstrated that wAIHA patients found the FACIT-Fatigue understandable, comprehensive, and relevant, and the data indicated that the instrument can detect a meaningful change in fatigue symptoms and impacts. Study findings support the content validity of the FACIT-Fatigue and contribute to the evidence that the FACIT-Fatigue is fit-for-purpose to evaluate fatigue in adults with wAIHA.

**Supplementary Information:**

The online version contains supplementary material available at 10.1186/s41687-025-00930-0.

## Background

Warm autoimmune hemolytic anemia (wAIHA) is a chronic, life-threatening disorder caused by autoantibodies that attach to and destroy red blood cells (RBCs) [8]. The term ‘warm’ designates the optimal temperature of 37 °C at which the antibodies react with RBC antigens [8]. Although wAIHA is the most common autoimmune hemolytic disease, the condition is very rare, diagnosed in 1–2 adults per 100,000 per year [15]. Prevalence of wAIHA has been estimated at 0.11 to 0.13 per 1000, or about 1 out of every 8000 people [5]. About half of cases are idiopathic and the remainder are associated with other medical conditions, such as certain malignant or rheumatological conditions, or exposure to certain drugs such as piperacillin or cephalosporins [15]. Symptoms of wAIHA are linked to hemolysis and the resulting anemia. These include fatigue, dyspnea, and palpitations [1], which can interfere with daily activities and reduce patients’ health-related quality of life. Clinical management of wAIHA can be challenging and severe disease is associated with a mortality rate of 4%-8% [9].

The rarity of wAIHA has limited the feasibility of executing randomized clinical trials to develop evidence-based recommendations for therapy [2] and there are no Food and Drug Administration-approved treatments specific to this condition [9]. Primary treatment for wAIHA typically involves a course of glucocorticosteroids, which is effective initially in about 80% of patients [2]. Patients with precipitous symptoms, severe hemolysis, or refractory or relapsing disease may also receive blood transfusions, treatment with intravenous immunoglobulin (IVIG) or rituximab, or undergo splenectomy or immunosuppressive therapy such as azathioprine, mycophenolate mofetil, or cyclosporine [2]. Many patients require ongoing low-dose steroid therapy to control wAIHA and these patients may experience chronic relapses of hemolysis that require repeat or alternate interventions [2]. Newer treatments under investigation in recent clinical trials include plasma-cell and B-cell targeting agents to reduce antibody production [2, 9]. Evaluation of treatment efficacy has long focused on hemoglobin response and prevention of relapses in hemolysis. However, recent clinical trials have also included assessment of patient-reported symptoms such as fatigue [9], including an ongoing trial (NCT04119050) of an investigational agent to block neonatal Fc receptor (FcRn) mediated recycling of antibodies to lower total antibody levels, including autoantibodies. Inclusion of patient-reported outcomes (PROs) in clinical trials provides a way to collect patient experience data and support evaluation of treatment benefit, but the instruments used to measure PROs must be fit-for-purpose and comport with regulatory guidance.

The Functional Assessment of Chronic Illness Therapy-Fatigue Scale (FACIT-Fatigue) is a 13-item PRO instrument (i.e., questionnaire) that takes less than five minutes to complete [7]. It includes five items about fatigue symptoms and eight items about impacts of fatigue on functioning. All items are formulated as statements (e.g., “I feel fatigued”). The instrument has a 5-category response scale that ranges from 0 (Not at all) to 4 (Very Much). The instrument is scored by reversing the item scores for all but two items, summing the item scores, multiplying by 13, and then dividing by the number of items answered. The resulting scores range from 0 to 52, with higher scores indicating less fatigue and better health-related quality of life. The instrument was developed in the 1990s to evaluate fatigue associated with anemia that develops in cancer patients [16]. Since its inception, the FACIT-Fatigue has been evaluated in several autoimmune health conditions [4, 12], but has not previously been evaluated for its content validity in the wAIHA patient population.

## Aim of this study

To assess the content validity of the FACIT-Fatigue in adults with wAIHA, this study aimed to evaluate comprehension, relevance, and individually-meaningful changes in score in a sample of wAIHA patients [14]. A patient-reported outcome questionnaire exhibits content validity when evidence demonstrates that it measures meaningful aspects of health that are relevant to the target patient population. Evidence of content validity contributes to the evaluation of an instrument’s fit-for-purpose as a clinical outcome assessment in each context of use.

## Methods

Two observational, cross-sectional studies were conducted to gather data through 60-minute, one-on-one qualitative interviews. Both interview designs included a concept elicitation section and cognitive debriefing of the FACIT-Fatigue items [3]. Concept elicitation for both studies focused on the symptoms of wAIHA and the impacts fatigue had on patients’ daily life. Study 1 debriefed the FACIT-Fatigue items, instructions and response options. Study 2 also debriefed the FACIT-Fatigue items and gathered additional data about patients’ perspectives on meaningful change in response for both improvement and worsening on each item. Cognitive debriefing included both a “think aloud” approach, in which participants provided unprompted commentary as they completed the instrument, and a follow-on discussion guided by interviewer probes intended to elicit data about item interpretation, relevance, missing content, ability to recall, and meaningful change in response. Participants in Study 2 were also asked to describe the relationship between each item concept and their overall assessment of fatigue severity.

Study 1 included a total of 10 qualitative telephone interviews with adult patients with wAIHA. Eligible participants had to be 18 years or older, diagnosed with wAIHA by a physician, currently experiencing fatigue believed to be related to wAIHA, and able to read and speak English. Study 2 included 10 additional qualitative video-conferencing interviews with patients with wAIHA. In addition to the eligibility criteria used for Study 1, participants were also required to have received their diagnosis of primary or secondary wAIHA at least 3 months prior to recruitment and to have received treatment for wAIHA. Employees and family members of employees of any of the collaborators involved with the project, individuals with significant comorbidities that might interfere with study objectives and those with any disorder that compromises ability to give informed consent were not eligible to participate. Study participants were recruited in the US via research partners using a variety of strategies (e.g., outreach via medical providers, advocacy groups, social media). All participants for both studies provided informed consent. Study 1 was reviewed by RTI IRB and approved on April 23, 2019, and Study 2 was reviewed and approved by Advarra CIRBI, a central Institutional Review Board (IRB) on November 3, 2022.

All interviews were audio-recorded and professionally transcribed verbatim, then de-identified for analysis. Descriptive statistics were used to characterize the study samples and identify patterns in the selection of item responses and, in the case of the second study, intervals of meaningful change. Inductive content coding was conducted to identify discrete concepts related to wAIHA symptoms and to the impacts associated with fatigue, and to record participant’s comments on comprehension and interpretation of the instrument’s questions and response options. Thematic analysis techniques were used to identify patterns across the data (e.g., bothersomeness). To develop a qualitative framework for meaningful change, the research team examined the frequency of point-change selections for each item across participants; within-participant consistency in point-change selections (e.g., consistently choosing one point changes vs. varying choice of point change); participants comments about response options they would never choose; and the ways participants described the relevance and meaning of each item, which helps determine which items are likely to change together because they address the same underlying experience within the fatigue construct (i.e., participants described items as similar in content, answered them in the same way, and selected the same point changes as meaningful). Meaningful change was not probed when an item was not relevant for a particular participant. Examples of ways that relevance and interpretation affected perspectives on meaningfulness of change in response included participants who regarded an item as a yes/no question; indicated that differences in response options were less clear for a specific item; said their ratings might hinge on factors other than wAIHA-related fatigue (e.g., ability to rearrange their plans or push through fatigue to complete usual activities); or indicated that and item was only relevant during some phases of illness (e.g., item An12, too tired to eat, was identified as relevant only for acute periods of anemia). Finally, analysis considered the reported relationships between fatigue symptoms (e.g., feeling tired, having energy) and functioning (e.g., ability to do usual activities) to understand whether people with wAIHA might report meaningful improvements for one but not the other. From these analyses, the research team then estimated the smallest aggregate change in response across all 13 items that would indicate a meaningful change in fatigue experience for each participant, irrespective of whether this point change reflected small changes across multiple items or a large change in response on a single item.

## Results

A total of 20 patients were enrolled, ten in each study, and interviews took place during 2019 and 2020 for Study 1 and 2022 and 2023 for Study 2. Demographic characteristics for the sample are summarized in Table 1. The mean age for participants was 47.5 ± 14.7 years, with a range of 23–70 years. Participants also resided in a wide range of US geographic locations.

**Table 1:** Participant demographic characteristics (*n* = 20)

Characteristic	Interview participants *n* (%)
**Sex**	
Female	11 (55)
Male	9 (45)
**Race/Ethnicity**	
Black or African American	2 (10)
Non-Hispanic White	16 (80)
Hispanic/Latino	2 (10)
**Time Since Diagnosis**	
Less than 1 year	4 (20)
1–5 years	10 (50)
More than 5 years	6 (30)
**Education**	
High school/GED	1 (5)
Some college	5 (25)
Completed college	14 (70)

During interviews, participants described 12 symptoms and five areas of impacts associated with wAIHA, as shown in Table 2. Of the 13 symptoms, fatigue was the only one reported by every participant, with all other symptoms reported by fewer than half the sample. Individual participants also described observable clinical signs such as blood in their urine, jaundice, pale skin, hypotension, swollen feet, or sweating.

**Table 2:** Symptoms and impacts reported by study participants during concept elicitation (*n* = 20)

Concept	Interview participants *n* (%)	Illustrative Quotations
**Symptoms**		
Fatigue	20 (100)	• It’s really difficult to describe…but it’s like a special kind of tired. It feels completely different than my normal tired, like any of the other tiredness. It’s unique. • I’m just drained, I have no energy. I feel like I’m 800 pounds…like I have cinderblocks weighing me down. It doesn’t matter how much I sleep, I’m just tired.
Dizziness, fainting	9 (45)	• Well, I was dizzy. I was lightheaded. I had trouble concentrating. I had blurred vision. I was confused. I felt like I was drunk, I guess, was really the only way I can explain it, I guess.
Weakness	7 (35)	• Where it’s literally a task to put one foot in front of the other or lift my arm up to put on a shirt. That’s how my weakness goes, like that’s how I can tell. It’s like, ‘Oh, okay. Well, definitely sit down.’ And it hurts almost.
Shortness of breath	6 (30)	• So when the disease was active, it was awful. I was short of breath, just sitting at my desk.
Trouble concentrating, confusion, brain fog	5 (25)	• I have to open up my calendar and say, ‘What did I do yesterday? What did I do two days ago? When was that appointment?’ I’m a little disoriented or brain-fogged when it comes to some of those things, and then other times I might be sharp as a tack.
Taste/smell changes, mouth pain	4 (20)	• I had weird like smell in my nose. • It’s not fun living with a mouth that feels like it, you just sucked on a scalded hot cup of coffee. It’s one of the most unpleasant things you can live with and it’ll turn anybody cranky. • And the other weird thing is that when you’re hemolyzing and you’re breaking out so many red blood cells…I could actually smell it, and I could taste it. And so my sense of taste changed.
Racing heart, chest pain	4 (20)	• My heart rate…being a runner, I wear a running watch that actually monitors my heart rate. Just sitting at my desk, my heart rate was right around 100 beats per minute.
Headaches	3 (15)	• Those two things [extreme tiredness, impaired vision] are like the first ones and a headache too…real intense headache.
Body or muscle aches	3 (15)	• You feel the ache in the muscles, the large core muscles from head to toe and your whole body aches. It wears you out, it wears you down and gives you fatigue is how I describe it.
Feel cold	2 (10)	• I also go through that coldness, be cold. And that’s when I get really tired, when I go through that cold stage.
Vision problems	2 (10)	• Well, I was dizzy. I was lightheaded. I had trouble concentrating. I had blurred vision. • It’s like it made my vision diminished. I saw black or little spots and stuff.
Pica	2 (10)	• And then like I also experiencing pica when I get it to like the number one thing that I notice is I’ll be sitting in the bathroom, going to the bathroom, and I will want to eat shampoo. And then when that happens, I’m like, ‘Oh, I should probably call the doctor.’ I know it’s really weird. I thought I was losing my mind the first time it happened. But then I like started like understanding anemia and what it can do and it just gives me pica.
**Impacts**		
Daily activities / Daily functioning	20 (100)	• I can’t do anything. I can’t go to the grocery store. I can’t walk around. Even things as simple as getting dressed and showering…I can’t do anything alone because I might pass out. • I don’t hardly do anything. I haven’t worked in years. I don’t really leave the house…I can’t really do anything like showering and cooking and cleaning…everything like that, I have to have help with. I live with my significant other and he has to assist me with pretty much…showering, dressing, cooking, things like that. • It affects every aspect of it. You wake up with an ache and that fatigue. You wake up feeling like you haven’t slept. And it takes a lot of effort…It takes twice as much energy or effort to lift a gallon of milk, and to shower, and to get dressed, and to function with your daily chores and going about your day. And then that ripples into your lifestyle and your activities and that reflects on your relationships with your loved ones. I’m a goal getter [sic], goal driven kind of person and I set myself up with reasonable goals and I can’t set goals anymore because all I do is set myself up for failure. I’ll set myself up for a goal to go to the farmers market on Saturday morning with my husband and spend… a couple of hours in the gorgeous sunshine and I can’t get the energy together to get dressed, to get out the door. Or to hang out and spend a couple of hours versus 15 min, I need to go, I’m tired. It affects every layer of my life. • It’s like I have to motivate myself even to try and to get in the shower when [the fatigue] is at its worse. It takes everything I have to just do simple things like that. I’ll just get something easy [to eat] because I literally do not have enough energy to make the food.
Emotional well-being	20 (100)	• It’s emotionally devastating…your brain wants to do one thing like as simple as take a shower or cook a meal and your body doesn’t have the physical ability to actually get it done. I think that just cycles into an emotional depression. • Some weeks I’m able to get everything done and it doesn’t bother me. And other weeks I can’t get anything done and I get very frustrated. • It’s depressing. It’s upsetting. It’s just not where I should be at 29 [years old]. I should be a mom. I should l have kids. I should be happy.
Work, school, or volunteer	16 (80)	• I was finding myself strategically putting sick days in so that I could have rest periods. Like if I knew that I had a busy week, I would plan a Friday off so that I would have a three-day weekend to recuperate. • When I feel fatigue very strong, I am not able to work. Since I work as a tutor [for children], I have to sometimes change my schedule and even cancel some classes. • [The fatigue is] devastating…here I am, I put myself through college while being a full-time soccer mom with two kids and a schedule at home. I’ve worked as a professional executive as a global human resource manager for 27 years. I know I have skill sets and things to offer companies…and here I am 5 years now effectively unemployed.
Social life/ Social functioning	16 (80)	• I mean when [the fatigue] happens, we can’t really predict it because there doesn’t seem to be a rhyme or reason…so if it’s a holiday, a birthday, we don’t participate, or everybody has to bring [the party] to me. • A perfect example is if I go out with friends. I end up being the first to leave and go home because I’m tired and I know I’ve got to get home and go to sleep and get enough rest so that I can be functional the next day. • It’s hard to be bubbly. It’s hard to be engaging. It’s hard to be present because you’re just so fatigued and weak. So just the idea of the social activity or social engagement with others is just daunting because it’s all the energy and effort to end up with a negative result. You just avoid it.
Need for rest/ sleep during the day	15 (75)	• I need to sleep during the day. That is a definite…I never like taking naps, but I’m forced to do them. My body just makes me. • I do what I can with the family, and they see that I’m having issues, and they see that I try to hide them, but sometimes they catch me sleeping in the back…I sit down for a minute and then lean back against the board and I fall asleep. Just give me a few seconds and I’m out.

All patients described fatigue as a prominent and bothersome symptom characterized by feelings of tiredness, need for daytime rest or sleep, and a pronounced lack of energy. Fatigue had substantial impacts on patients’ daily functioning, including reduced ability to work or go to school, difficulty taking care of routine daily responsibilities, disruptions to family and social life, and negative impacts on emotional well-being. Severe episodes of fatigue were debilitating, sometimes leaving participants house- or bed-bound. Mild chronic fatigue was also bothersome and burdensome, requiring many patients to organize their lives around the need for naps and rest periods and limiting their capacity to work or engage in other meaningful activities. This chronic fatigue, along with the uncertainty about potential flares in symptoms and impacts, was also mentally and emotionally draining.

Participants reported impacts on daily functioning that spanned activities of daily living (e.g., bathing, dressing) as well as routine activities such as taking care of children or pets. Impacts on ability to work, volunteer, or go to school ranged from needing to take occasional sick days to full disability. Most participants reported regularly needing to rest or sleep during the day, which resulted in additional impacts on these aspects of daily life. Participants described the symptoms and impacts of wAIHA as emotionally devastating, with frustration and depression frequently mentioned. Finally, most participants described impacts on their ability to engage in normal social or family life, which included avoidance of social activities. The unpredictability of fatigue and other wAIHA symptoms contributed to these perceived impacts because participants found it difficult to anticipate whether a planned activity could be completed at all or would leave them feeling drained. As one participant described debilitating fatigue that could strike at any time: “I definitely have good days and bad days…and I never know when I’m going to have a bad day. [On a bad day] pretty much just curled up, either watching TV or in bed. A good day is when I’m out running errands, getting things done, doing some volunteer work, just being active, walking my dogs.”

**Fig. 1:**
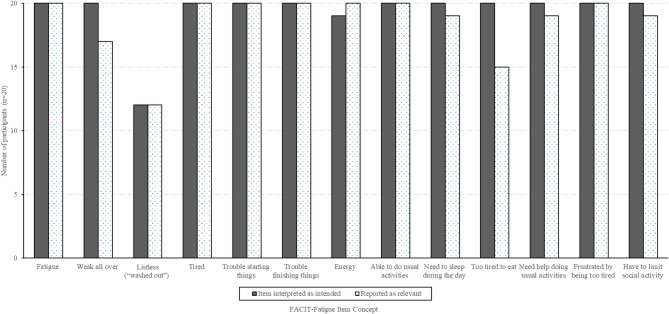
Interpretation and relevance of FACIT-Fatigue items among interview participants (*n* = 20)

Fatigue-related concepts identified during interviews are clearly reflected in the FACIT-Fatigue items, and interview results indicated that the FACIT-Fatigue was readable, well understood, comprehensive, and relevant to individuals with wAIHA (Fig. 1; quotations illustrating participant understanding and views on relevance are provided in the online supplementary material). The item An1 referencing the terms “listless” and “washed out” was not well understood by 40% of participants. These participants were unfamiliar with these terms and did not correctly infer their meaning. For example, one participant stated: “Washed out gets a little bit of the mental fatigue I was talking about, maybe the sort of brain fog. But it’s hard to know really clearly what that means, honestly.” Several other participants thought these terms meant “pale” or “lack of color.” Study participants were easily able to recall their experiences over a 7-day period and rate them with the available response options, and did not indicate that different or additional response options were needed. Most participants did not identify any missing fatigue-related concepts; one participant identified fatigue upon exertion (e.g., while exercising) and mental fatigue as possible missing concepts. A few participants indicated that they found some questions repetitive, but also noted that they could still appreciate a distinction between each item (e.g., items asking about ‘fatigue,’ ‘tired’, and ‘energy’).

Most participants who discussed meaningful change (Study 2) perceived clear differences between each response option and could relate the available options to their own experiences with wAIHA. In making judgments about meaningfulness of change, participants referenced both incremental differences in the frequency with which experiences occurred as well as the severity of symptoms or the degree of impairment of function (see online supplementary material for additional quotes). For example, one participant commented for item H17 (feel fatigued):

It [a 1-point improvement in response] would be very meaningful…I would be much more active, I’d have more energy, I would be able to go out and get those seeds planted in the garden that need to be done, like today. Like I said, I’m pretty active. I’d probably be out there riding right now, riding my mule. Just, it would be a lot of changes. The house would be a little dusted.

Across items, most participants identified a 1-point change in response as representative of a meaningful change for both improvement and worsening (Table 3). Participants associated these incremental changes with tangible differences in their daily experiences, as described by one individual in addressing the meaningfulness of 1-point changes in response to item H17 (feel fatigued):

Yeah, so if it did go [from mild] to none, I would see that as a very meaningful improvement because I would be feeling fully energized…If it changed from mild to moderate, I would not be feeling great. I would be probably getting less things done in my daily life and just not being as present as I would want to be.

In Study 2, six of ten participants identified a change of more than one point as representative of meaningful change for at least one item; only one participant did so consistently for all items. In most cases, changes of more than one point involved moving all the way to the end of the scale (i.e., to ‘not at all’ or ‘very much’) and appeared to reflect the participant’s desire for a full resolution of fatigue symptoms or impacts. One participant, for example, stated that for change on item H17 (feel fatigued) to be meaningful, “Well, you know, I think I would have to stop feeling the extreme fatigue altogether, I mean, honestly.”

**Table 3:** Participant selections of meaningful change by item (*n* = 10)

Item	1 point (*n*)	More than 1 point (*n*)	Meaningful change not probed (*n*)*
I feel fatigued	7	3	-
I feel weak all over	7	1	2
I feel listless (“washed out”)	6	2	2
I feel tired	5	4	1
I have trouble starting things because I am tired	5	2	3
I have trouble finishing things because I am tired	4	4	2
I have energy	7	3	-
I am able to do my usual activities	6	3	1
I need to sleep during the day	7	2	1
I am too tired to eat	4	3	3
I need help doing my usual activities	7	2	1
I am frustrated by being too tired to do the things I want to do	7	2	1
I have to limit my social activity because I am tired	6	2	2

*Meaningful change was generally not probed when an item was not relevant to a given participant (e.g., participants who had never experienced being too tired to eat, or who stated that they were not sociable or would not start things they could not finish)

The interview data supported the development of a hypothesized qualitative framework for meaningful change. A minimum 3-point change in total score when all items are rated likely reflects a meaningful change in fatigue at the individual patient level for anyone with wAIHA, for either improvement or worsening, while score changes below this threshold appear unlikely to be meaningful in any context. This framework was founded in the following observations of the data:

1. Five of the 13 FACIT-Fatigue items (HI7 ‘feel fatigued,’ An2 ‘feel tired,’ An5 ‘have energy,’ An7 ‘able to do usual activities,’ and An8 ‘need to sleep during the day’) appeared to be overlapping or aligned, with participants providing similar interpretations of the meaning and opinions of the relevance of these items. These items were very strongly associated with the overall severity of fatigue and participants identified them as central to measuring benefits from treatment. As a result, these items appear capable of reflecting changes in experience across all participants and across the full spectrum of fatigue experiences. However, two of these items (An7, An8) were influenced by coping choices for some participants, lending support for a change threshold of 3-points rather than 5-points. For example, some participants indicated that they would push through their fatigue to complete usual activities, so they responded that they were able to do them even though it was challenging and exhausting to do so. Similarly, some participants indicated that they often felt like they wanted to sleep during the day, but did not “need to” (a phrase included in the item wording). They noted instead that they tended to push through and avoid napping unless their fatigue was very severe.
2. Three items (HI12 ‘feel weak all over,’ An12 ‘too tired to eat,’ and An14 ‘need help doing my usual activities’) were interpreted as reflecting greater levels of fatigue and appeared to be highly relevant to people with wAIHA when they were most severely affected. These results suggest that single-point changes in response to these three items may be useful for detecting meaningful changes in experience for people who are most severely ill, who might report improvements and decrements in these items even if responses to other items do not change.
3. Two items also seem capable of producing a meaningful 3-point change in response selection on their own. For example, participants who experienced times when they were too tired to eat (An12) described this as a short-term experience that was binary in nature: you either are, or are not, too tired to eat. Participants did not perceive meaningful increments of being too tired to eat, suggesting that they might shift between ‘Very much’ and ‘Not at all,’ and that use of the intermediate ratings might be less common depending on the frequency of assessment. Similarly, a 3-point change in needing help doing usual activities (An14) would constitute a meaningful change in overall fatigue severity even if other ratings did not change.

## Discussion

Fatigue is a key symptom of wAIHA that has significant impacts on patients’ health-related quality of life and ability to function. It is important to assess patients’ self-report of fatigue status using a valid assessment instrument alongside clinical indicators when evaluating treatment efficacy. Study participants understood the FACIT-Fatigue instrument and had no difficulty recalling and rating their experiences with fatigue over the 7-day reference period. Some participants found the item about feeling “listless” and “washed out” confusing, a finding that has also surfaced in recent studies of the FACIT-Fatigue in other patient populations [6, 10], but all other items were consistently interpreted as intended. The “listless/washed out” terminology could reflect the vernacular of the US Midwestern region in the mid-1990s when the original patient samples for item development and validation were obtained [16], thus item revision may be warranted to improve consistency of respondent comprehension in future. However, this single item may also make minimal or no difference for score interpretation as it was well-understood by most participants.

The hypothesized minimum meaningful change threshold of 3 points is aligned with thresholds found in other qualitative and quantitative studies of FACIT-Fatigue in populations with other chronic health conditions that are characterized by fatigue [4, 11, 13]. The study findings indicate that this threshold would apply equally well to patients with wAIHA who are experiencing a range of fatigue severity levels, either episodically or chronically. Psychometric assessment of the instrument to evaluate its measurement properties, including anchor-based methods for evaluating meaningful change, is an important next step to confirm that the FACIT-Fatigue scale is fit-for-purpose in the adult wAIHA patient population.

## Limitations

The studies’ limitations include the small sample sizes and limited diversity of participants’ demographic characteristics, as well as the restriction to US-resident, English-speaking individuals. Study participation was limited to individuals who reported having experienced fatigue due to wAIHA. All interviews began with an open-ended question intended to elicit spontaneous information (e.g., “Could you tell me what it’s like to have wAIHA?”) but then focused on fatigue. These elements of the study design may have limited the studies’ capacity to elicit data about other important wAIHA symptoms or impacts. Three of the 10 participants in Study 2 described themselves as in remission and experiencing only mild symptoms associated with wAIHA. To address this limitation, participants were asked to describe both their current experiences and how they would have responded to the instrument items in the past when their wAIHA symptoms were more severe.

## Conclusion

Results from these concept elicitation and cognitive debriefing interviews provide evidence supporting the content validity of the FACIT-Fatigue for adults with wAIHA. Study participants found the FACIT-Fatigue to be comprehensive and relevant to their fatigue-related experiences with wAIHA.

## Supplementary Information

Below is the link to the electronic supplementary material.

Supplementary Material 1

## Data Availability

The datasets generated and/or analyzed during the current studies are not publicly available due the potential for whole transcripts to be used to re-identify participants, but may be available from the corresponding author on reasonable request.

## Abbreviations

wAIHA: Warm autoimmune hemolytic anemia
FACIT: Functional Assessment of Chronic Illness Therapy
IRB: Institutional Review Board
PRO: Patient-reported outcome
RBC: Red blood cells
