# Supplementary material for: Content validity and meaningful change for the FACIT-Fatigue scale in warm autoimmune hemolytic anemia: results from qualitative interview studies with patients
Source: J Patient Rep Outcomes. 2025 Jul 29;9:97. doi: 10.1186/s41687-025-00930-0 (PMC12307827; doi:10.1186/s41687-025-00930-0)
Supplement: Supplementary file 1 — Supplementary Material 1 [file 41687_2025_930_MOESM1_ESM.docx]

**Supplementary Material #1: Quotations Illustrating Item Comprehension and Perspectives on Meaningful Change for FACIT-Fatigue in adults with wAIHA**

| **Item #** | **Item concept** | **Quotes illustrating participants’ item interpretation and views on relevance** | **Quotes illustrating perspectives on meaningful change in response (improvement or worsening)** |
| --- | --- | --- | --- |
| H17 | Fatigue | - ‘I feel fatigued,’ I think [this question] was the first and most important one to ask. - That I feel too tired to do the things that I either need to do or I want to do. But in my mind, I feel like I just want to lay down, that I just can't finish what I'm working on at the moment. | - It would be very meaningful. It would be a miracle. I would be much more active, I'd have more energy, I would be able to go out and get those seeds planted in the garden that need to be done, like today. (1 point improvement) - I have to go to sleep, like I feel like I'm about to fall asleep on the wheel type of feeling, or like, you know, I'm doing everything, like getting 8 hours, getting 8, 10 hours of sleep but I wake up tired after doing the dishes or like I sleep multiple times a day (2 point worsening) |
| HI12 | Weak all over | - I mean, for me, "weak all over" is like, it's too much work to lift your arms, to go places. Like, you really just want to melt into furniture. - Extreme fatigue to me has always been very hard to describe to somebody that hasn't experienced extreme fatigue in this way. So I haven't really felt weak all over. ... It's just, it's very hard to explain. It's just like I feel like lead, you know. I feel like I just can't move my muscles. It's like all of your muscles are not wanting to function, you know. Your legs just – you have to, like, really focus to pick your legs up. I'll trip sometimes when I'm – like, because I'm walking, and I'm just really focused. And I'm trying to move my legs forward. And so, I would say it's not really a weak all over. | - So, basically, it just depends on how much physical strength I have. That's how I view it, like, "Do I have the physical strength to climb a set of stairs or the physical strength to lift something?" Like, the good is like, "Okay, I feel normal. I can do everything," right? The extreme end of it is, "Oh, like, I can't even pick up this thing, or like climbing a flight of stairs leaves me breathless." (1 point improvement/worsening) - For the meaningful worsening, I would say it would be quite a bit for this one, and that would be when probably my anemia is active, really causing me to not fulfill a lot of tasks. (2 point worsening) |
| An1 | Listless (“washed out”) | - It can be not every day but some days, I feel very, very listless or washed out…very weak. Not doing anything. Cannot do my chores. Cannot do work. - Listless, washed out. I'm trying to imagine what that even means different from all the other questions you've asked. I mean, that actually – yeah, that would've described me in the evenings when I didn't know that I had it yet and it wasn't being treated. …actually, the "washed out" gets a little bit of the mental fatigue I was talking about, maybe the sort of brain fog. But it's hard to know really clearly what that means, honestly. | - Definitely, meaning that I would be able to schedule the classes. If I was more energetic – if I did not have the fatigue, I would be able to schedule the classes. (1 point improvement) - For me, you know, it's kind of exhausted overall, maybe, and I definitely don't feel exhausted overall, although if it got to where it was "somewhat," then that would definitely be something I'd be concerned about. (1 point worsening) |
| An2 | Tired | - The tiredness is—usually is one of the first problems. Like, I stay in bed a little longer and things like that. - Well, I think tired and fatigued are a little bit two different things because that I could be tired because I hadn't slept well or be tired because my mind was tired from working, you know. Fatigue to me is a physical thing, where tired could be a mental tired or a physical tired, so I think that was the difference in my answer, you know, because I feel I'm more fatigued than I am tired, if that makes sense. ... fatigued is more the right word to me because like I said, tired could be, you know, you ran a marathon or, you know, what I'm saying, you know, because you're tired. You know, fatigued to me is a more internal health issue. | - I mean "not at all," because I'd be thinking I was feeling really good, it would be. But, the lines between "a little bit" and "somewhat," I mean, that can kind of change – you know, almost – well, it's going to change, depending on what activities are happening, you know? (2 point improvement) - So me being somewhat tired, I would say that's – basically, like, I'm able to still do my everyday things, but maybe I'm not as motivated in them. So they're not me working or doing them at, maybe, a 100 percent, more at, like, an 80 percent. (1 point worsening) |
| An3 | Trouble starting things | - I wake up and I get up and wash dishes or something. Then I sit there and I say, ‘Maybe I’ll start it in about an hour. Let me lay here for a little longer.’ So it’s like you’re not getting up, doing things. You’ve got to push yourself up to do it. - The way I interpret the question is, you know, I don't have the energy to do anything, like to start any goals or tasks. | - If I'm more tired, then I'm seriously going to look at all the options for "What am I going to start?" you know? "Am I so tired that doing an activity in the chair is not even an option?" or is it just something more simple like, "You know what? You really don't need to go to the store today. You can wait until tomorrow," [chuckles] or something like that. I: And, if you were to answer "not at all" instead of "a little bit," would that represent a meaningful improvement in your fatigue? A: Yes, because if I'm not feeling tired at all, pretty much, if I have the thought to go do it, I'm probably going to go do it. (1 point improvement) - "Somewhat" is like I have to dig for motivation. (1 point worsening) |
| An4 | Trouble finishing things | - And it’s usually the same thing [as trouble starting things], only the other end of it. Because I’ll get tired halfway through doing something and [think] ‘I’ll finish it tomorrow.’ - I'm going to say not at all. I start a job, and I finish it. I'm just – again, that's part of my personality. Even if it takes me longer, which it does more now than it did, I'm going to finish what I started. | - I'm just thinking scenarios in my life that I have started and just called it a day before it was finished and then [audio cuts out] finished it the next day. If I was just a little bit tired, I might have just squeezed it out and finished it, but somewhat tired, that means I'm done for the day. It'll be there for tomorrow. (1 point improvement/worsening) - Basically for this one, I kind of put myself in what tasks I do in my every day, so in my – different tasks have different levels of fatigue that they can cause. So if I'm saying, like, I started working on, say, in my life, a blog post, and I wasn't able to finish it and I wanted to finish it, I would say that would be a little bit. Where something like cooking, if I'm in the middle of cooking, and I can't finish cooking, I see that as more severe of something because you don't just put cooking aside. And I would classify that more of a somewhat. (2 point worsening) |
| An5 | Energy | - I have energy. So for the past 7 days, I would do a 1, a little bit…I’ve been able to get dressed sometimes by myself. I have a service dog, so I’ve been able to take him potty. I’ve been able to put food down for him. I’ve been able to do small things by myself. Little things that didn’t require assistance. - When I think of energy, it's a little different from, like, how tired you are. I'm thinking of my ability to do tasks, so do I have the energy to, basically, if I have a list of things I want to do today, do I have the energy to complete them all. | - It would have to be like I have a lot of energy or I have a moderate amount of energy. I: All right, so you said "somewhat." So, if you said "quite a bit," would that be a meaningful improvement? A: Yeah, yeah. I mean "quite a bit," yeah. It's meaningful, yes. I: Okay, and to represent a meaningful worsening of your fatigue, how much would your answer for this question have to change? A: So, like no energy, like zero. I: Mm-hmm. "Not at all?" A: Yeah, "not at all." (1 point improvement, 2 point worsening) - It would mean that I'm eating better and that I'm feeling better and more motivated to – energy, you know, energy sometimes to me can come from your mind too, you know, and what you're doing. It can be a mental thing as well, but, you know, I would say a little bit would mean that I'm feeling better…At this point, I'm not really looking to have – I'm just glad to feel better. (1 point improvement) |
| An7 | Able to do usual activities | - It’s just the normal day-to-day things around the house, and appointments, and grocery shopping, and things like that. - You know, getting up, getting ready for work, working all day, you know, working eight hours, and, you know, that takes a lot of brain cells. And then going home, dinner, you know, getting the family straight. I have animals, so taking care of animals. That's my usual activities. | - It would go to a little bit, you know, because, again, that's a driven person, you know, pushing themselves to do things that they probably shouldn't be doing, you know, but they're doing them anyways. So I would say worsening would be moving down to a little bit. (1 point worsening) - It would be huge. I would be able to get so much more done without being so tired. (1 point improvement) |
| An8 | Need to sleep during the day | - I never like taking naps, but I’m forced to do them. My body just makes me. - The way I interpret it is like, "Is my fatigue so bad that I need to sleep?" I put "not at all" just because, like, yeah, I want to but it's not bad enough where I have to. …I mean, I think it's extremely relevant just because it just shows like how tired I am, you know, like how it's to the degree at which I have to rest in order just to do other things again. - I'm not a person that takes naps, even when I'm tired. | - I would say it would go to not at all, yeah, because I didn't used to need a nap, you know. I didn't feel – so, like, I needed to, like, rejuvenate myself or rest myself. I just didn't do that. My sister used to say you're the only one that takes naps. Yes. Like, why? I need them, you know, so I would say not at all. (2 point improvement) - Yeah, I mean, you know, it kind of sounds crazy, but with the anaemia, one of the things is, seriously, "Where am I on a napping scale?" "Am I just kind of tired and I just need to sit down.? Am I full-blown tired and I need to take a nap, and by the way, we're picking up something from out for dinner, you know?" And, if I am that tired, I do not care if it is five o'clock when I get off work – I'm taking a nap, and the nap is going to be an hour and a half long. (1 point improvement, 2 point worsening) |
| An12 | Too tired to eat | - When the fatigue hits me, I just can't eat. And when I was in the middle of it, I mean, I was almost, you couldn't even chew, you were so tired. But now, like, this was in the last seven days, correct? - I pretty much, well, always eat. The day that I had the severe anemia and went to the hospital for it and didn’t know I was that sick, I literally ate a few hours before, so I just – that one doesn't really affect me. I will always eat despite my fatigue. | - Would probably be a little bit. I'm not a huge eater, and I eat small meals, so I would say a little bit would be meaningful improvement. (1 point improvement) - I think for me, the difference between "A little bit" and "Somewhat" is so minute. I would have to say "Quite a bit" to give you a meaningful answer that I was too tired to eat. "A little bit" is, eh, I don't have that much of an appetite. I'll eat it, but it doesn't really appeal to me today. We all eat food that doesn't appeal to us. That's not normal for me. I like food. I like to eat. So, this was a different thing for me, not – having an indifference whether I eat or not. (2 point worsening) |
| An14 | Need help doing usual activities | - Matter of fact, I'd get offensive [sic] when somebody wanted to help me. People wanted to bring me food, and it's like, "Thank you but no thank you." I didn't need – that's not me. - I mean, when I was fully anemic, I would need help. I had to move back in with my parents during the summer. I was deemed – my doctors said I could not live by myself. Food, laundry, walking around, people needed to be next to me. There was very little that I could do by myself. | - A meaningful improvement would be not at all. I'd be able to do things totally by myself, and hopefully I'll get there. (3 point improvement) - Because I selected not at all, I would say a little bit for this one, so having to ask for anything, I would say that would be meaningful. (1 point worsening) |
| An15 | Frustrated by being too tired | - Some weeks I’m able to get everything done and it doesn’t bother me. And other weeks I can’t get anything done and I get very frustrated. - Again, when I have a responsibility to do, it gets done without any delay. I'm never late on a project that's due or anything like that. But not being able to go out socially because I know that I'm either going to be tired early in the evening or that I don't want to go in the first place because I've already had a full day type thing, that definitely gets frustrating because that's not who I was prior to it | - Not at all, not frustrated at all… It plays an important role, like I said, because it's just a completely frustrating illness in, like I said, that it just takes so much away from you. It's frustrating in a lot of ways. (3 point improvement) - I mean, I guess you could improve on it by it being "not at all," but I don't know. I mean, really, there are still probably going to be things that are going to frustrate me because I'm still probably going to be triggered one way or another. Or, something else is going to frustrate me and I'm not realizing that it's not necessarily tiredness – it's something else, you know? But, if we were going to then go to something else, it would probably be "quite a bit," and it would then be pretty severe. (1 point improvement, 2 point worsening) |
| An16 | Have to limit social activity | - It’s hard to be bubbly. It’s hard to be engaging. It’s hard to be present because you’re just so fatigued and weak. So just the idea of the social activity or social engagement with others is just daunting because it’s all the energy and effort to end up with a negative result. You just avoid it. - Well, I'm not a big social person anyway, and so I think that's why I said a little bit because it's taken some of my social activities away, but I wasn't really social before that. | - I would say just move it to not at all, to be able to just do what, you know, socially what you want to do and go where you want to go. (1 point improvement) - I would, in order for me to start decreasing my active social activity, it would have to probably be either "somewhat" or "quite a bit," and I'm leaning more towards "quite a bit" because, you know, I'm going to have to be really, pretty much, pretty tired before I'm like, "Nope, I don't even think I could do it," because I would still at least go out and attempt to do it. (2-3 point worsening) |
